# Single-crystal X-ray diffraction and NMR crystallography of a 1:1 cocrystal of di­thia­non and pyrimethanil

**DOI:** 10.1107/S2053229617000870

**Published:** 2017-02-06

**Authors:** Ann-Christin Pöppler, Emily K. Corlett, Harriet Pearce, Mark P. Seymour, Matthew Reid, Mark G. Montgomery, Steven P. Brown

**Affiliations:** a Department of Physics, University of Warwick, Coventry CV4 7AL, United Kingdom; b Department of Organic Chemistry, University of Würzburg, 97074 Würzburg, Germany; c Molecular Analytical Science Centre for Doctoral Training, University of Warwick, Coventry CV4 7AL, United Kingdom; d International Research Centre, Syngenta, Jealott’s Hill, Bracknell, Berkshire RG42 6EY, United Kingdom; e Afton Chemical, London Road, Bracknell, Berkshire RG12 2UW, United Kingdom

**Keywords:** NMR crystallography, solid-state NMR, di­thia­non, pyrimethanil, cocrystal, hydrogen bonding, C—H⋯π inter­actions, fungicides

## Abstract

A single-crystal X-ray diffraction structure of a 1:1 cocrystal of two fungicides, namely di­thia­non (DI) and pyrimethanil (PM), is reported [systematic name: 5,10-dioxo-5*H*,10*H*-naphtho­[2,3-*b*][1,4]dithiine-2,3-dicarbo­nitrile–4,6-dimethyl-*N*-phenyl­pyrimidin-2-amine (1/1), C_14_H_4_N_2_O_2_S_2_·C_12_H_13_N_2_]. Following an NMR crystallography approach, experimental solid-state magic angle spinning (MAS) NMR spectra are presented together with GIPAW (gauge-including projector augmented wave) calculations of NMR chemical shieldings. Specifically, experimental ^1^H and ^13^C chemical shifts are determined from two-dimensional ^1^H–^13^C MAS NMR correlation spectra recorded with short and longer contact times so as to probe one-bond C—H connectivities and longer-range C⋯H proximities, whereas H⋯H proximities are identified in a ^1^H double-quantum (DQ) MAS NMR spectrum. The performing of separate GIPAW calculations for the full periodic crystal structure and for isolated mol­ecules allows the determination of the change in chemical shift upon going from an isolated mol­ecule to the full crystal structure. For the ^1^H NMR chemical shifts, changes of 3.6 and 2.0 ppm correspond to inter­molecular N—H⋯O and C—H⋯O hydrogen bonding, while changes of −2.7 and −1.5 ppm are due to ring current effects associated with C—H⋯π inter­actions. Even though there is a close inter­molecular S⋯O distance of 3.10 Å, it is of note that the mol­ecule-to-crystal chemical shifts for the involved sulfur or oxygen nuclei are small.

## Abstract 2

A combined single-crystal X-ray diffraction and NMR crystallography study of a 1:1 cocrystal of two fungicides, namely di­thia­non and pyrimethanil, is presented. Specifically, the role of hydrogen bonding and C—H⋯π and S⋯O inter­molecular inter­actions is qu­anti­tatively investigated.

## Introduction   

With an increasing global population, limited availability of arable land, an increase in extreme weather events and growing pest resistance to certain existing agrochemical products, innovation in the agrochemical industry is as important as ever if we are to provide enough food for everyone. With lower usage rates, ease of use and more favourable toxicology profiles being important objectives, the search for and structure-based design of potential agrochemical products needs to become more efficient (Lamberth *et al.*, 2013 ▸). One possibility in this regard is the usage of cocrystals formed between an active ingredient and coformers or other active ingredients *via* reversible noncovalent inter­actions. While this is an established procedure in the development of new active pharmaceutical ingredients, where it is used to increase the solubility and bioavailability (Blagden *et al.*, 2007 ▸), there is also great potential to exploit cocrystals in the optimization and development of agrochemicals. For example, a reduced solubility could increase the agrochemical’s residence time on the respective plant and multicomponent entities could improve the release profile (and thus absolute usage), as well as allow the simultaneous delivery of two or more active components. However, the design of suitable cocrystalline materials and prediction of their properties and formed cocrystal structures is far from being trivial. Some design strategies based on the hierarchy of inter­molecular inter­actions (Aakeroy & Salmon, 2005 ▸) or the assessment of the solubilities and saturation temperatures of the pure compounds to be included in a cocrystalline arrangement (ter Horst *et al.*, 2009 ▸) are available as a guideline. However, if multiple and different hydrogen-bonding donors and acceptors are present in the mol­ecules, a reliable prediction of the resulting structure becomes very difficult (Bhatt *et al.*, 2009 ▸).

NMR crystallography, namely the combination of experimental solid-state magic angle spinning (MAS) NMR with calculation of NMR parameters, is finding important application to moderately sized organic mol­ecules (Harris, 2004 ▸; Elena *et al.*, 2006 ▸; Harris *et al.*, 2009 ▸; Bonhomme *et al.*, 2012 ▸). We present here an NMR crystallography analysis of the 1:1 cocrystal of two fungicides, namely di­thia­non (DI) and pyrimethanil (PM). Specifically, following a preparation protocol in Sowa *et al.* (2013 ▸), a single-crystal X-ray diffraction structure determination is reported, with this structure (after DFT geometry optimization) providing the input for a calculation, using the GIPAW (gauge-including projector augmented wave) method (Pickard & Mauri, 2001 ▸; Yates *et al.*, 2007 ▸), of the NMR chemical shieldings. The computational analysis is complemented by the recording of 1D (one-dimensional) and 2D (two-dimensional) experimental ^1^H and ^13^C MAS NMR spectra. Building upon studies of pharmaceutical cocrystals by such an NMR crystallography investigation (Tatton *et al.*, 2013 ▸; Dudenko *et al.*, 2013 ▸; Stevens *et al.*, 2014 ▸; Kerr *et al.*, 2015 ▸; Sardo *et al.*, 2015 ▸; Luedeker *et al.*, 2016 ▸), we present here the application of this approach to an agrochemical cocrystal.

## Experimental and computational details   

### Sample preparation   

The DI–PM cocrystal was prepared according to method VII in point [0041] of Sowa *et al.* (2013 ▸), *i.e.* dry di­thia­non and pyrimethanil (both solids) were mixed thoroughly in a 1:1 molar ratio (0.5 g of pyrimethanil) and kept at 323 K under agitation. After a couple of hours, the powdery product had changed to a dark-olive-green colour.
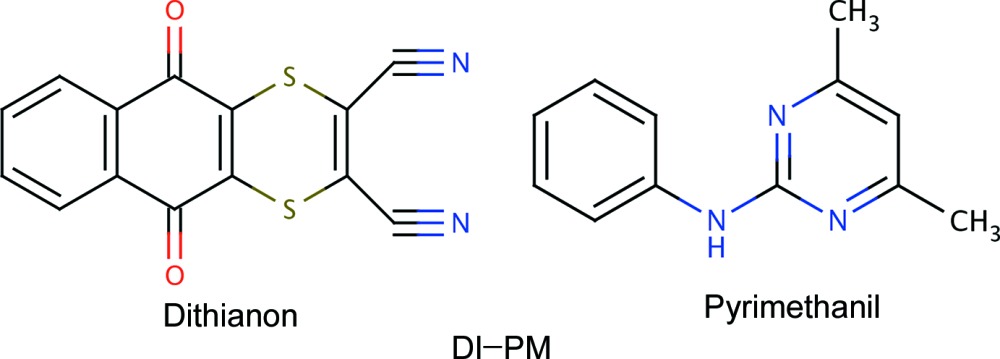


### Single-crystal X-ray diffraction: structure solution and refinement   

Crystal data, data collection and structure refinement details are summarized in Table 1 ▸. The H atoms were all located in a difference map, but those attached to C atoms were repositioned geometrically. The H atoms were initially refined with soft restraints on the bond lengths and angles to regularize their geometry [C—H = 0.93–0.98 Å and N—H = 0.86–0.89 Å, and with *U*_iso_(H) = 1.2–1.5*U*_eq_(parent)], after which the positions were refined with riding constraints (Cooper *et al.*, 2010 ▸).

### Solid-state NMR   

1D ^1^H MAS and 1D ^13^C cross polarization (CP) MAS experiments were performed on a Bruker Avance III spectrometer operating at ^1^H and ^13^C Larmor frequencies of 600 and 150.9 MHz, respectively, using a 1.3 mm HXY (^1^H MAS) or a 4 mm HX (^13^C CP MAS) Bruker probe. In all cases, a ^1^H 90° pulse duration of 2.5 µs was used. 2D ^1^H–^13^C HETCOR experiments were performed on a Bruker Avance III spectrometer, using a 4 mm HXY probe in double-resonance mode. In the HETCOR pulse sequence, the following phase cycling was employed: ^1^H 90° pulse (90° 270°), ^13^C 180° pulse (2{0°} 2{180°}), ^13^C CP contact pulse (4{0°} 4{180°} 4{90°} 4{270°}), receiver (0° 180° 0° 180° 180° 0° 180° 0° 90° 270° 90° 270° 270° 90° 270° 90°). For CP, a 70 to 100% ramp (Metz *et al.*, 1994 ▸) on the ^1^H channel was used for the CP contact time. During acquisition of a ^13^C FID, SPINAL64 (Fung *et al.*, 2000 ▸) ^1^H heteronuclear decoupling was applied with a pulse duration of 5.9 µs at a nutation frequency of 100 kHz. A 2D ^1^H DQ experiment with BABA recoupling (Sommer *et al.*, 1995 ▸; Schnell *et al.*, 1998 ▸) was performed on a Bruker Avance III spectrometer operating at a ^1^H Larmor frequency of 700 MHz using a 1.3 mm HXY Bruker probe. A 16-step phase cycle was used to select Δ*p* = ±2 on the DQ excitation block and Δ*p* = −1 on the *z*-filter 90° pulse, where *p* is the coherence order. In all 2D experiments, the States–TPPI method was used to achieve sign discrimination in *F*_1_. ^13^C and ^1^H chemical shifts are referenced with respect to TMS using l-alanine at natural abundance as an external reference: 177.8 ppm for the ^13^C carboxyl­ate resonance and 1.1 ppm for the ^1^H methyl resonance. All experiments were performed at room temperature, though frictional effects due to MAS increase the actual sample temperature (Langer *et al.*, 1999 ▸).

### DFT calculations   

Calculations were performed using *CASTEP* (Clark *et al.*, 2005 ▸; Academic Release Version 8.0) and employed the PBE exchange-correlational functional (Perdew *et al.*, 1996 ▸). For both geometry optimization and NMR shielding calculations, a plane-wave basis set with ultrasoft pseudopotentials (Vanderbilt, 1990 ▸) with a maximum plane-wave cut-off energy of 700 eV was used. A Monkhorst–Pack grid of minimum sample spacing 0.05 × 2π Å^−1^ was used to take integrals over the Brillouin zone. Geometry optimization was performed with the unit-cell parameters fixed, starting from the single-crystal X-ray structure. The positions of the 208 atoms in the unit cell (*Z* = 4, *Z*′ = 1) were relaxed and periodic boundary conditions were applied. The space group *P*2_1_/*n* was preserved. All distances and angles stated in the main text of this article are for the geometry-optimized crystal structure. Note also that the geometry optimization within *CASTEP* causes a relabelling of the atoms – in this article, we use the *CASTEP* numbering; see Fig. S1 in the *Supporting information* for a comparison with the numbering employed in the crystallographic CIF file. The GIPAW method (Pickard & Mauri, 2001 ▸; Yates *et al.*, 2007 ▸) was utilized for the NMR chemical-shielding calculations, which were performed on the geometry-optimized structure. For the isolated mol­ecule calculations, a single mol­ecule (either DI or PM) from the fully geometry optimized structure is kept in the unit cell, whose dimensions are also increased by ∼5 Å in each direction – the NMR shieldings are then calculated without any further geometry optimization.

## Results and discussion   

### Single-crystal X-ray diffraction structure   

**Figure 1:**
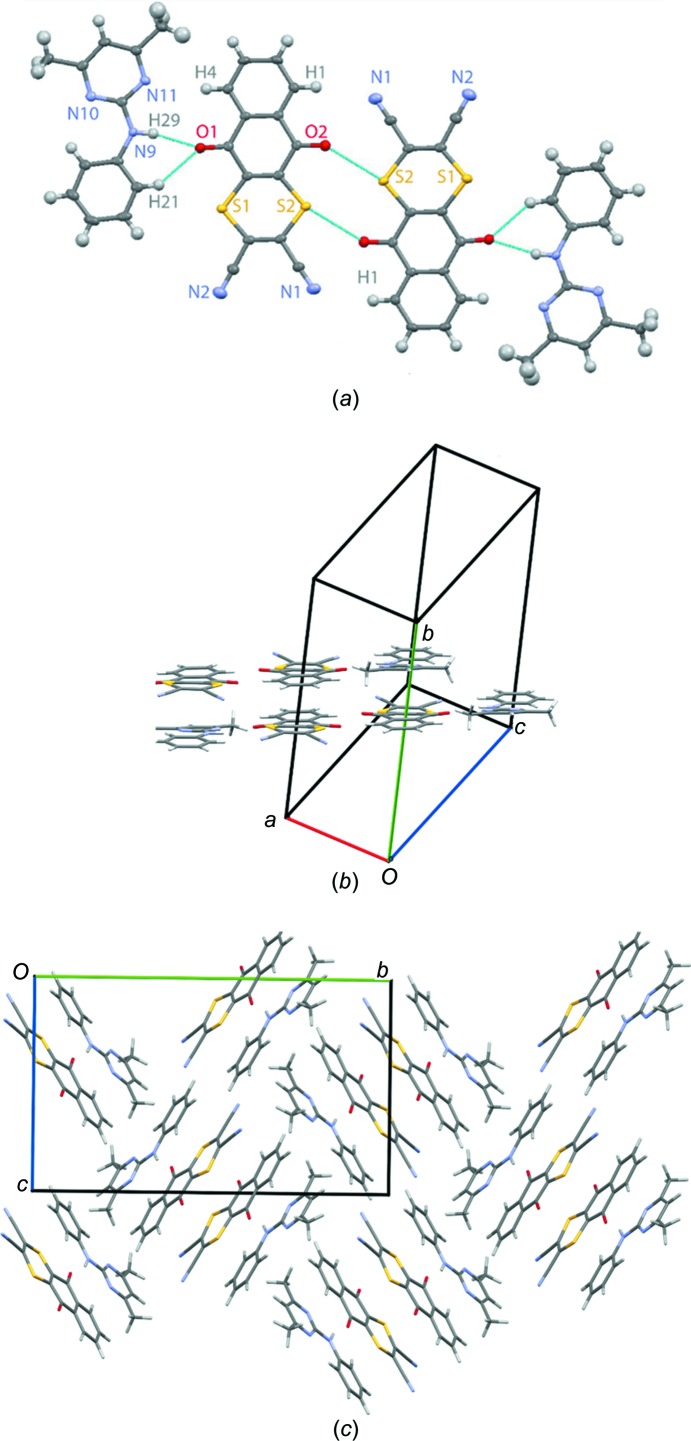
Representations of the crystal structure of the DI–PM cocrystal, showing (*a*) the inter­molecular inter­actions within a ‘chain’ of mol­ecules, with displacement ellipsoids drawn at the 50% probability level, (*b*) the packing of two chains of mol­ecules as ‘layers’ and (*c*) the ‘zigzag’ arrangement of chains (viewed along the crystallographic *a* axis). In parts (*b*) and (*c*), the unit cell is shown, indicating the *a*, *b* and *c* unit-cell axes.

The single-crystal X-ray diffraction structure of the DI–PM cocrystal is schematically represented in Fig. 1 ▸. As shown in Fig. 1 ▸(*a*), a chain of mol­ecules is held together by N—H⋯O and C—H⋯O hydrogen bonds (between DI and PM mol­ecules) and by putative S⋯O inter­actions (Burling & Goldstein, 1992 ▸) between two DI mol­ecules; note that the relative strengths of these inter­actions is investigated below (see §3.5) using GIPAW calculations of NMR chemical shieldings. The further packing of two chains of mol­ecules as ‘layers’ and a ‘zigzag’ arrangement of chains are shown in Figs. 1 ▸(*b*) and 1(*c*), respectively. As can be seen from the representation along the crystallographic *a* axis in Fig. 1 ▸(*c*), the packing is based on assemblies of blocks of four mol­ecules; four mol­ecules (PM–DI–DI–PM) are arranged in a layer (Fig. 1 ▸ *a*), forming a block that is perpendicular to an adjacent block of four mol­ecules, thus building up the ‘zigzag’ arrangement.

### Experimental and calculated ^13^C chemical shifts   

**Figure 2:**
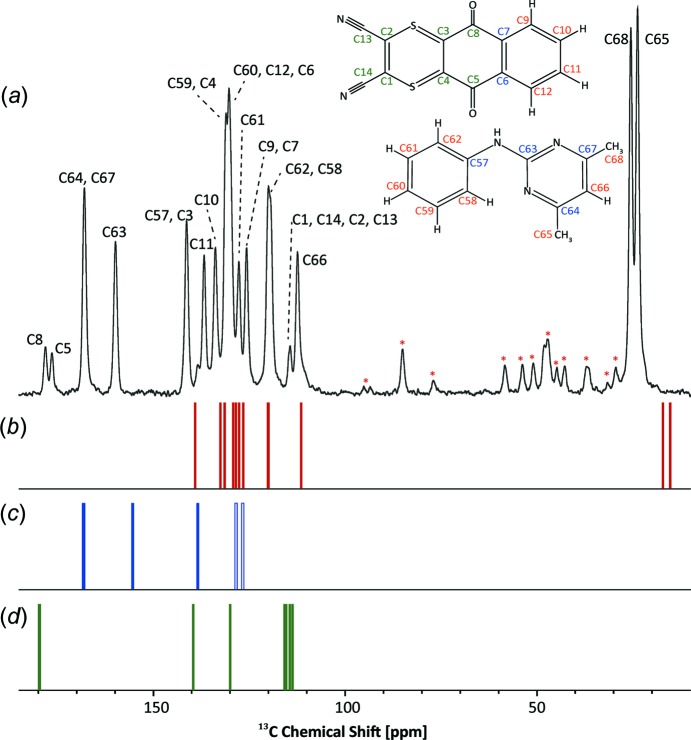
(*a*) A ^1^H (600 MHz)–^13^C CP MAS (12.5 kHz) NMR spectrum of the DI–PM cocrystal (* denote spinning sidebands), together with (*b*)–(*d*) stick spectra corresponding to calculated (GIPAW) ^13^C chemical shifts (see Table 2 ▸). Separate stick spectra are presented according to whether correlation peaks corresponding to (*b*) direct C—H bonds or (*c*) longer-range C⋯H proximities are observed in the ^1^H–^13^C 2D spectra presented in Fig. 4 ▸, or (*d*) where no experimental correlation peaks are observed. In the CP MAS experiment, a contact time of 1.4 ms was used and 1024 transients were co-added for a recycle delay of 57 s.

Fig. 2 ▸ presents a ^13^C CP MAS NMR 1D spectrum (Fig. 2 ▸ *a*) of the DI–PM cocrystal, together with three stick spectra (Figs. 2 ▸ *b*, 2*c* and 2*d*) that represent ^13^C chemical shifts calculated using the GIPAW method for the DI–PM crystal structure. Specifically, the calculated ^13^C chemical shifts are presented in three groups according to whether they correspond to direct one-bond C—H connectivities (Fig. 2 ▸ *b*, red labels) or nonprotonated C atoms (Figs. 2 ▸ *c* and 2*d*, blue and green labels, respectively). The distinction between Figs. 2 ▸(*c*) and 2(*d*) corresponds to whether cross peaks corresponding to a longer-range C⋯H proximity are observed in ^1^H–^13^C 2D correlation spectra (see §3.4).

### One- and two-dimensional ^1^H MAS NMR spectra   

**Figure 3:**
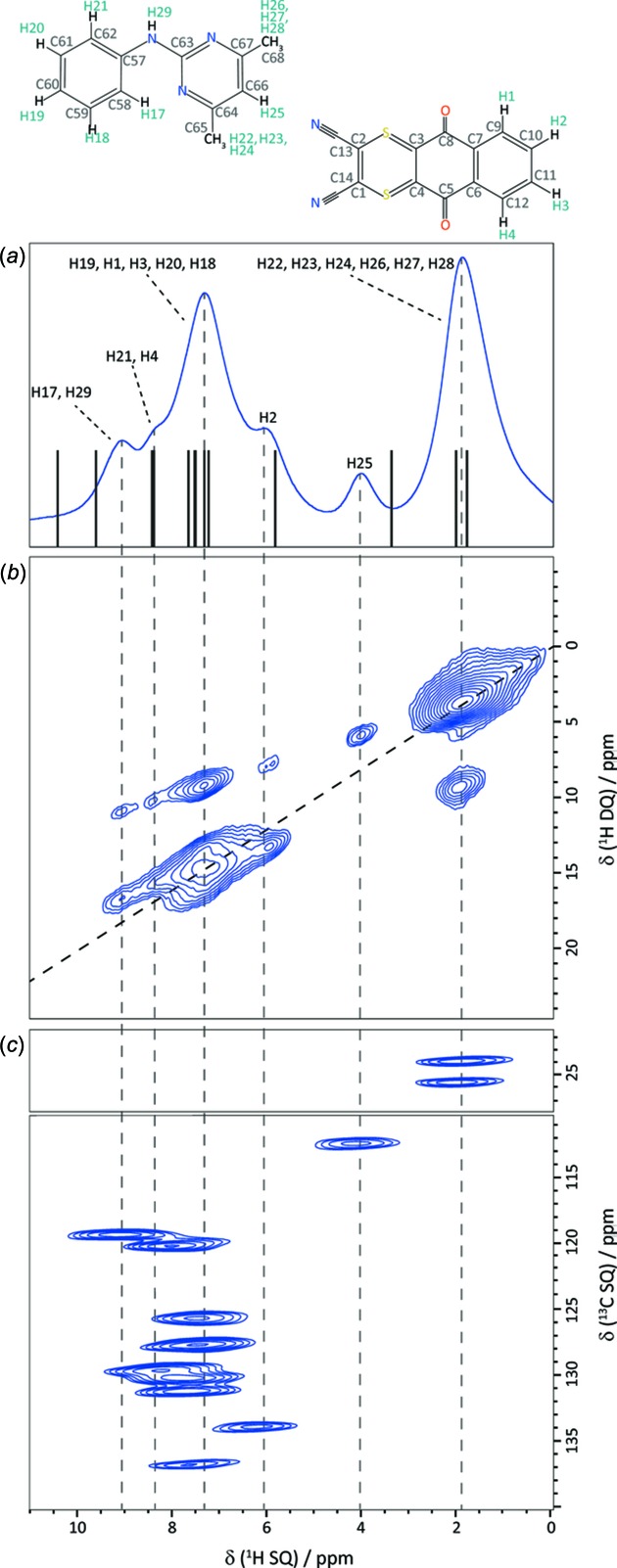
MAS NMR spectra of the DI–PM cocrystal, showing (*a*) a ^1^H (600 MHz) MAS (60 kHz) one-pulse spectrum (16 transients were co-added for a recycle delay of 15 s), (*b*) a 2D ^1^H (700 MHz) DQ MAS (60 kHz) spectrum (the dashed diagonal line indicates the *F*_1_ = 2*F*_2_ DQ–SQ diagonal) recorded using one rotor period of BABA recoupling (32 transients were co-added for each of 200 *t*_1_ FIDs using a recycle delay of 6 s, corresponding to a total experiment time of 12 h) and (*c*) a ^1^H (500 MHz)–^13^C HETCOR MAS (12.5 kHz) spectrum recorded using FSLG ^1^H homonuclear decoupling in *t*_1_ and a short CP transfer duration of 100 µs (104 transients were co-added for each of 128 *t*_1_ FIDs using a recycle delay of 6 s, corresponding to a total experimental time of 22 h). The vertical lines in part (*a*) correspond to calculated (GIPAW) ^1^H chemical shifts. For the ^1^H–^13^C NMR spectrum in part (*c*), two separate spectral regions are presented corresponding to methyl and aromatic C—H groups; note that this spectrum has been rotated through 90° from its usual representation [the ^13^C dimension corresponds to direct (*t*_2_) acquisition]. The base contour level is at (*b*) 7% and (*c*) 20% of the maximum peak height.

Figs. 3 ▸(*a*) and 3(*b*) present ^1^H NMR spectra of the DI–PM cocrystal recorded at a fast MAS frequency of 60 kHz; specifically, a one-pulse one-dimensional spectrum in Fig. 3 ▸(*a*), together with vertical lines corresponding to calculated (GIPAW) ^1^H chemical shifts, as well as a 2D DQ spectrum in Fig. 3 ▸(*b*). In addition, Fig. 3 ▸(*c*) presents a ^1^H–^13^C 2D correlation spectrum of the DI–PM cocrystal; note that this spectrum has been rotated through 90° from its usual representation such that the direct (^13^C) dimension is vertical. In this way, it is possible to directly compare (see vertical dashed lines) ^1^H chemical shifts of peaks in the ^1^H–^13^C (Fig. 3 ▸ *c*) and ^1^H DQ 2D (Fig. 3 ▸ *b*) and ^1^H 1D (Fig. 3 ▸ *a*) spectra. Two separate spectral regions are presented in Fig. 3 ▸(*c*) corresponding to (top) the methyl resonances at a ^13^C chemical shift close to 25 ppm and (bottom) the aromatic CH resonances with ^13^C chemical shifts between 110 and 140 ppm.

The ^1^H–^13^C correlation spectrum in Fig. 3 ▸(*c*) was recorded using a short CP contact time of 100 µs to transfer magnetization from ^1^H to ^13^C, such that cross peaks correspond to one-bond C—H connectivities. The spreading of the resonances into two dimensions in Fig. 3 ▸(*c*) allows the identification of two and ten resolved cross peaks for the CH_3_ and aromatic CH groups, respectively. The value of such a ^1^H–^13^C correlation spectrum in resolving and assigning the experimental ^1^H chemical shifts is thus evident. Table 2 ▸ lists the calculated (GIPAW) and experimental ^13^C chemical shifts (sorted in order of increasing chemical shift). For directly bonded C—H connectivities, H-atom labels and calculated (GIPAW) and experimental ^1^H chemical shifts are presented in normal font.

**Figure 4:**
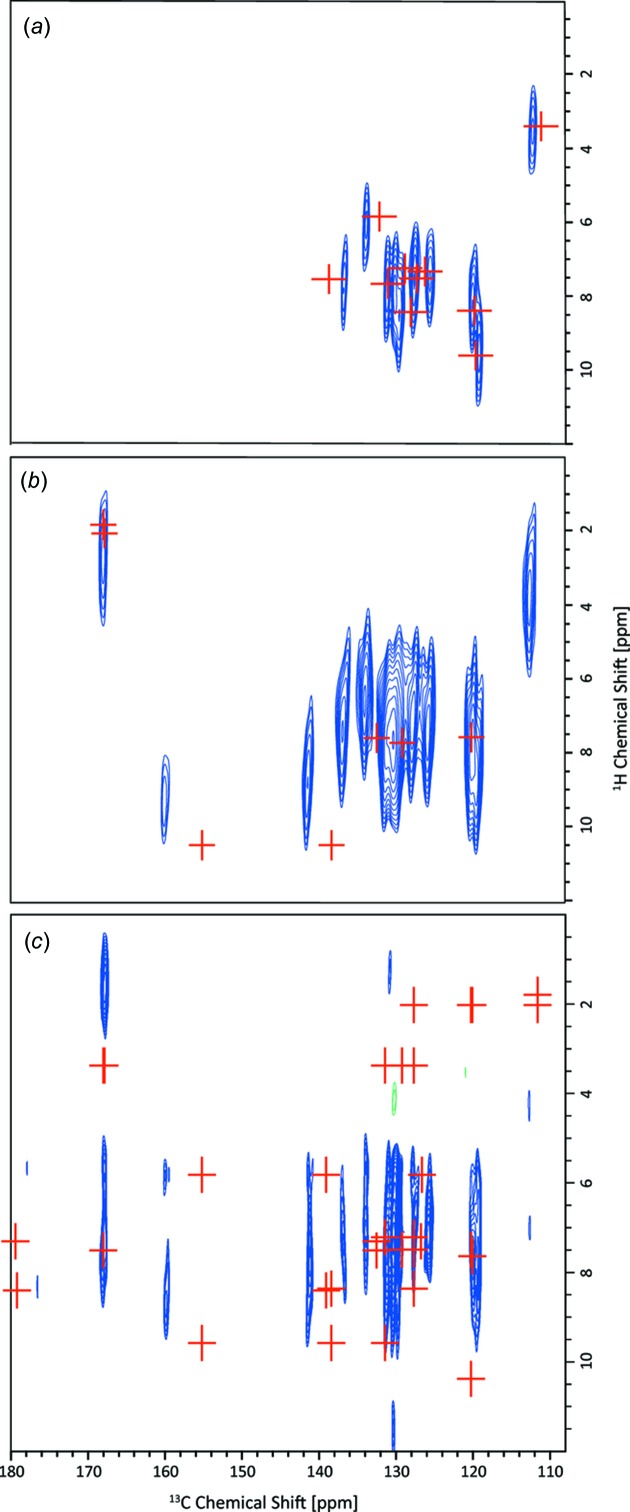
^1^H (500 MHz)–^13^C HETCOR MAS (12.5 kHz) spectra of the DI–PM cocrystal recorded using FSLG ^1^H homonuclear decoupling (Bielecki *et al.*, 1989 ▸) in *t*_1_ with a CP transfer duration of (*a*) 100 µs, (*b*) 500 µs and (*c*) 1 ms. The spectrum in part (*a*) is repeated from Fig. 3 ▸(*c*). 104 transients were co-added for each of (*b*) 128 or (*c*) 90 *t*_1_ FIDs using a recycle delay of (*b*) 6 or (*c*) 5.5 s, corresponding to a total experimental time of (*b*) 22 or (*c*) 14 h. The scaling factor in *F*_1_ was determined to be (*a*) and (*b*) 1.80 or (*c*) 1.73. The base contour level is at (*a*) 20, (*b*) 13 and (*c*) 25% of the maximum peak height. Red crosses correspond to GIPAW-calculated ^1^H and ^13^C chemical shifts (see Table 2 ▸) for (*a*) one-bond C—H bonds and (*b*) and (*c*) C⋯H proximities between (*b*) 1.2 and 2.2 Å, and (*c*) 2.2 and 3.0 Å.

Fig. 4 ▸ compares ^1^H–^13^C correlation spectra recorded with three different CP contact times of 100 µs (Fig. 4 ▸ *a*), 500 µs (Fig. 4 ▸ *b*) and 1 ms (Fig. 4 ▸ *c*); Fig. 4 ▸(*a*) is a copy of Fig. 3 ▸(*c*), but presented in the normal orientation, *i.e.* with the direct (^13^C) dimension horizontal. It is evident that additional cross peaks are observed for longer CP contact times – these correspond to longer-range C⋯H proximities (see italics font in Table 2 ▸). Notably, cross peaks are observed at ^13^C chemical shifts of 141.5 (atom C57), 160.1 (atom C63) and 168.2 ppm (atoms C64 and C67); these all correspond to intra­molecular proximities within the di­thia­non mol­ecule, *i.e.* C57 with H17 (9.1 ppm, 2.16 Å), H21 (8.0 ppm, 2.16 Å) and H29 (9.1 ppm, 2.06 Å), C63 with H29 (9.1 ppm, 2.01 Å), C64 and C67 with H25 (4.0 ppm, 2.16 and 2.17 Å) and CH_3_ protons (1.9 and 2.0 ppm, nearest distance 2.14 Å). Of most inter­est is the (160.1 ppm, 9.1 ppm) cross peak, which thus enables the determination of the NH ^1^H chemical shift.

With all the ^1^H chemical shifts assigned, let us re-examine the ^1^H DQ MAS spectrum in Fig. 3 ▸(*b*). In such a spectrum, cross peaks are observed in the DQ dimension at the sum of the two single-quantum (SQ) frequencies if there is a close proximity (typically up to 3.5 Å; Brown, 2007 ▸, 2012 ▸) between the corresponding two H atoms (a full listing of H⋯H proximities under 3.5 Å for the DI–PM cocrystal is given in Table S1 of the *Supporting information*). Consider the two lowest-ppm aromatic CH protons H25 (4.0 ppm) and H2 (6.2 ppm) for which distinct ^1^H resonances are resolved in the ^1^H SQ dimension. For H25, the only DQ peak is at 4.0 + 2.0 = 6.0 ppm with the CH_3_ protons, since H25 is sandwiched between two methyl-group substituents on the PM mol­ecule. For H2, there is a DQ peak at 6.2 + 7.5 = 13.7 ppm corresponding to the intra­molecular H⋯H proximity with the neighbouring H1 (7.4 ppm, 2.50 Å) and H3 (7.7 ppm, 2.47 Å) DI aromatic CH protons, as well as a DQ peak at 6.2 + 2.0 = 8.2 ppm due to inter­molecular proximities to the PM CH_3_ H atoms (H23, H24, H28 and H22 at 2.90, 3.03, 3.12 and 3.12 Å, respectively). Considering the high-ppm region, DQ cross peaks for the overlapping PI NH H29 (9.1 ppm) and aromatic CH H17 (9.1 ppm) resonances are observed at 9.1 + 7.7 = 16.8 ppm for intra­molecular H29⋯H21 (2.21 Å) and H17⋯H18 (2.50 Å) proximities, as well as at 9.1 + 2.0 = 11.1 ppm for inter­molecular proximities to PM methyl-group protons (closest distances of H17⋯H26 = 2.48 Å and H29⋯H24 = 2.64 Å). For the other overlapping CH aromatic resonances, cross peaks due to intra­molecular proximities with other CH aromatic resonances, as well as inter­molecular proximities to the methyl protons, are also observed.

### Comparison of experimental and calculated ^1^H and ^13^C chemical shifts   

In the ^1^H–^13^C correlation spectra presented in Fig. 4 ▸, red crosses correspond to calculated (GIPAW) ^13^C and ^1^H chemical shifts. Specifically, in Fig. 4 ▸(*a*), red crosses correspond to direct C—H one-bond connectivities (C—H distances under 1.2 Å), while in Figs. 4 ▸(*b*) and 4(*c*), red crosses are presented for C—H proximities between 1.2 and 2.2 Å (Fig. 4 ▸ *b*), and between 2.2 and 3.0 Å (Fig. 4 ▸ *c*). We comment here on the level of agreement between experimental and calculated (GIPAW) chemical shifts. Starting with a consideration of the aromatic CH moieties (see Fig. 4 ▸ *a* and Table 2 ▸), the discrepancy between experiment and calculation is within 2 ppm for the ^13^C chemical shifts (except for C11, where the difference is 2.4 ppm); this corresponds to the established observation that the discrepancy is within 1% of the chemical shift range (∼200 ppm for ^13^C chemical shifts of diamagnetic mol­ecules). For the ^1^H chemical shifts, while most are within the usual 0.3 ppm, some exhibit slightly larger discrepancies, notably 0.6 ppm for atoms H17 and H25.

For the two CH_3_ groups (see Figs. 2 ▸ and 3 ▸ *a*, and Table 2 ▸), there is excellent agreement for the ^1^H chemical shifts (within 0.1 ppm), whereas the calculated ^13^C chemical shifts are both 8.5 ppm lower than the experimental values, although the experimental difference in ^13^C chemical shifts between atoms C65 and C68 of 1.8 ppm is reproduced by the calculation (difference of 1.9 ppm). The explanation for this is well known, namely, the gradient of a plot of experimental ^13^C chemical shifts against calculated shielding deviates slightly from −1 (Harris *et al.*, 2007 ▸; Ashbrook & McKay, 2016 ▸), such that calculated ^13^C chemical shifts are too low and too high compared to experiment for low-ppm and high-ppm resonances if, as here (see Fig. 2 ▸), the gradient is constrained to −1 and a single reference shielding is used. An alternative approach would be to use different reference shieldings for different regions of the spectrum (Webber, Emsley *et al.*, 2010 ▸).

Returning to the ^1^H chemical shifts, the biggest discrepancy is for the NH proton (H29), where the calculated ^1^H chemical shift of 10.5 ppm is 1.4 ppm higher than the experimental value of 9.1 ppm. Such a large difference is explained by a known temperature dependence (the experimental ^1^H chemical shift increases upon reducing the temperature) for hydrogen-bonded protons (Brown *et al.*, 2001 ▸; Pickard *et al.*, 2007 ▸; Webber, Elena *et al.*, 2010 ▸), considering that the calculation corresponds to 0 K.

### Calculated mol­ecule-to-crystal changes in chemical shifts   

**Figure 5:**
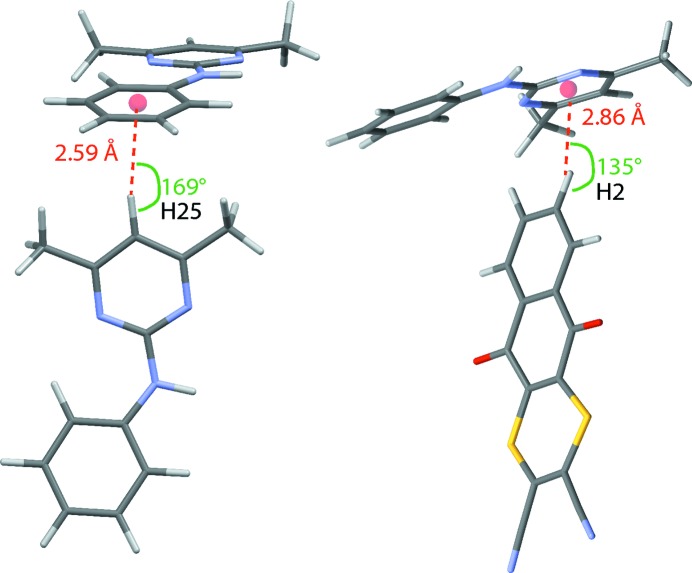
Schematic representations showing C—H⋯π inter­actions for aromatic atoms (*a*) H25 and (*b*) H2.

For cases such as the DI–PM cocrystal in this article, an NMR crystallography study is able to provide new insight by means of a comparison of chemical shifts calculated for the full crystal structure with those calculated for an isolated mol­ecule (as extracted from the geometry-optimized crystal structure) (Yates *et al.*, 2005 ▸; Schmidt *et al.*, 2006 ▸; Mafra *et al.*, 2012 ▸). Specifically, a mol­ecule-to-crystal difference in chemical shift is indicative of a combination of inter­molecular inter­actions, notably hydrogen bonding and ring currents due to C—H⋯π inter­actions, whereby the latter can be separately qu­anti­fied by means of the nucleus independent chemical shift (NICS) (von Ragué Schleyer *et al.*, 1996 ▸; Sebastiani, 2006 ▸; Uldry *et al.*, 2008 ▸; Mafra *et al.*, 2012 ▸). Consider Table 3 ▸, which presents the change in ^1^H chemical shift upon going from an isolated mol­ecule to the full crystal, Δδ_crystal–mol­ecule_, for the different H atoms in the DI–PM cocrystal. The largest positive change of 3.6 ppm is observed for the NH (H29) atom that is involved in an inter­molecular N—H⋯O hydrogen bond to atom O1 (see Fig. 1 ▸ *a*; the N⋯O and H⋯O distances are 2.95 and 1.96 Å, respectively, with a 162° N—H⋯O angle). Inter­estingly, Δδ_crystal–mol­ecule_ = 2.0 ppm for the aromatic CH H21 atom, for which Fig. 1 ▸(*a*) identifies an inter­molecular C—H⋯O so-called weak hydrogen-bonding (Desiraju & Steiner, 1999 ▸; Yates *et al.*, 2005 ▸; Uldry *et al.*, 2008 ▸) inter­action (the C⋯O and H⋯O distances are 3.24 and 2.35 Å, respectively, with a 138° C—H⋯O angle). The other H atoms, for which the magnitude of Δδ_crystal–mol­ecule_ exceeds 1 ppm, are H25 (−2.7 ppm) and H2 (−1.6 ppm); as shown in Fig. 5 ▸, these marked changes in the ^1^H chemical shift are a consequence of ring current effects associated with the proton pointing towards the centre of a six-membered aromatic ring of a nearby PM mol­ecule in a C—H⋯π inter­action, as has been noted previously in a number of other cases (Brouwer *et al.*, 2008 ▸; Mafra *et al.*, 2012 ▸; Brown, 2012 ▸).

In the above discussion in §3.1, a close S⋯O distance, equal to 3.10 Å, between the O2 and S2 atoms of neighbouring DI mol­ecules was noted; this is less than the sum of the van der Waals radii (3.32 Å) (Beno *et al.*, 2015 ▸; Zhang *et al.*, 2015 ▸). Indeed, there is a growing literature discussing S⋯O inter­actions (Burling & Goldstein, 1992 ▸; Iwaoka *et al.*, 2002 ▸; Beno *et al.*, 2015 ▸). While we have not carried out ^17^O or ^33^S solid-state NMR experiments as part of this study, an NMR crystallography approach enables the effect of such a putative S⋯O inter­action on the oxygen and sulfur NMR chemical shieldings to be investigated by means of the GIPAW calculation that reports on all nuclei in the solid-state structure. An inspection of Table 4 ▸ shows that it is inter­esting that Δδ_crystal–mol­ecule_ (note that this is the negative of the difference in calculated absolute shielding, with the latter being stated in Table 4 ▸) is much larger for O1 (−98 ppm), which is involved in a N—H⋯O inter­molecular hydrogen bonding, as compared to that for O2 (−23 ppm). Moreover, the change for S2 (13 ppm) is less than that for S1 (25 ppm), with both changes being small, though there is limited information on the range of experimentally observed solid-state NMR ^33^S chemical shifts (Hansen *et al.*, 2008 ▸). We conclude that even though there is a close inter­molecular S⋯O distance of 3.10 Å in the DI–PM cocrystal, there is not a marked effect on the calculated NMR chemical shieldings for the O2 and S2 nuclei.

## Summary   

In summary, we have presented here an NMR crystallography study of an agrochemical cocrystal. Specifically in combination with a GIPAW calculation of the NMR shieldings, ^1^H–^13^C 2D correlation spectra enable the resolution and assignment of the NH, aromatic CH and methyl resonances for the DI–PM cocrystal, while specific intra- and inter­molecular H⋯H proximities are identified in a ^1^H DQ MAS spectrum. The performing of separate GIPAW calculations for the full crystal structure and isolated DI and PM mol­ecules yields the change in the NMR chemical shift upon going from the mol­ecule to the crystal structure, thus allowing the qu­anti­tation of specific N—H⋯O, C—H⋯O and C—H⋯π inter­actions.

## Supplementary Material

Crystal structure: contains datablock(s) global, I. DOI: 10.1107/S2053229617000870/df3006sup1.cif

Structure factors: contains datablock(s) I. DOI: 10.1107/S2053229617000870/df3006Isup3.hkl

CASTEP cif output. DOI: 10.1107/S2053229617000870/df3006sup2.txt

magres file. DOI: 10.1107/S2053229617000870/df3006sup4.txt

Additional Tables (DQ NMR data and distances as well as a comparison of experimental and calculated (GIPAW) 13C chemical shift values) and a Figure showing the difference in the numbering schemes between the crystallographic data and the output of the GIPAW (CASTEP) calculations. DOI: 10.1107/S2053229617000870/df3006sup5.pdf

CCDC reference: 1507863

## Figures and Tables

**Table 1:** Experimental details

Crystal data	
Chemical formula	C_14_H_4_N_2_O_2_S_2_·C_12_H_13_N_3_
*M*_r_	495.59
Crystal system, space group	Monoclinic, *P*2_1_/*n*
Temperature (K)	100
*a*, *b*, *c* (Å)	7.1707 (2), 22.8006 (6), 13.8237 (4)
β (°)	97.047 (3)
*V* (Å^3^)	2243.04 (7)
*Z*	4
Radiation type	Cu *K*α
μ (mm^−1^)	2.45
Crystal size (mm)	0.60 × 0.10 × 0.02

Data collection	
Diffractometer	Agilent Xcalibur Onyx Ultra
Absorption correction	Multi-scan (*CrysAlis PRO*; Agilent, 2014 ▸)
*T*_min_, *T*_max_	0.596, 1.000
No. of measured, independent and observed [*I* > 2.0σ(*I*)] reflections	5143, 3160, 2667
*R*_int_	0.035
θ_max_ (°)	58.9
(sin θ/λ)_max_ (Å^−1^)	0.556

Refinement	
*R*[*F* ^2^ > 2σ(*F* ^2^)], *wR*(*F* ^2^), *S*	0.045, 0.094, 0.98
No. of reflections	3141
No. of parameters	109
No. of restraints	3
H-atom treatment	H atoms treated by a mixture of independent and constrained refinement
Δρ_max_, Δρ_min_ (e Å^−3^)	0.43, −0.37

Computer programs: *CrysAlis PRO* (Agilent, 2014 ▸), *SUPERFLIP* (Palatinus & Chapuis, 2007 ▸), *CRYSTALS* (Betteridge *et al.*, 2003 ▸), *CAMERON* (Watkin *et al.*, 1996 ▸) and *Mercury* (Macrae *et al.*, 2006 ▸).

**Table 2:** Comparison of calculated (GIPAW)^*a*^ and experimental ^13^C and ^1^H NMR chemical shifts (in ppm) in the DI–PM cocrystal^*b*^

Atom label		^13^C		^1^H	
C	H	δ_calc_	δ_expt_	δ_calc_	δ_expt_
C65	H22/H23/H24^*c*^	15.3	23.9	1.8	1.9
C68	H26/H27/H28^*c*^	17.2	25.7	2.0	2.0
C66	H25	111.5	112.6	3.4	4.0
C1	–	113.8	114.4^*d*^	–	–
C14	–	114.5	114.4^*d*^	–	–
C2	–	115.5	114.4^*d*^	–	–
C13	–	115.9	114.4^*d*^	–	–
C58	H17	120.1	119.4	9.7	9.1
C62	H21	120.2	120.3	8.4	8.0
C9	H1	126.7	125.7	7.4	7.4
C7	*H1^*e*^*	126.8	125.7	*7.4*	*7.4*
C61	H20	127.7	127.7	7.6	7.4
C12	H4	128.5	129.8	8.5	8.2
C6	*H4^*e*^*	128.6	129.8	*8.5*	*8.2*
C60	H19	129.3	130.2	7.3	7.8
C4	–	130.1	131.1^*d*^	–	–
C59	H18	131.5	131.2	7.7	7.7
C10	H2	132.6	133.9	5.9	6.2
C11	H3	139.2	136.8	7.6	7.7
C57	*H21, H17, H29*	138.5	141.5	*8.4, 9.7, 10.5*	*8.9*
C3	–	139.7	141.4^*d*^	–	–
C63	*H29*	155.5	160.1	*10.5*	*9.1*
C67	*H26/H27/H28, H25*	168.2	168.2	*2.0, 3.4*	*2.8*
C64	*H22/H23/H24, H25*	168.4	168.2	*1.8, 3.4*	*2.8*
C5	–	179.7	176.5^*d*^	–	–
C8	–	179.9	178.2^*d*^	–	–

Notes: (*a*) calculated isotropic chemical shifts are determined from calculated chemical shieldings according to δ_calc_ = σ_ref_ − σ_calc_, where σ_ref_ equals 30.0 ppm for ^1^H and 163.2 ppm for ^13^C. (*b*) H-atom labels and calculated and experimental ^1^H chemical shifts are presented in normal font for direct one-bond C—H connectivities, while longer-range C⋯H proximities (corresponding to cross peaks observed in the ^1^H–^13^C spectra presented in Figs. 4 ▸ *b* and 4 ▸ *c*) are presented in italics. (*c*) For CH_3_ groups, the calculated ^1^H chemical shifts correspond to the average over the three H atoms. (*d*) Experimental chemical shifts taken from the ^13^C CP MAS spectrum (Fig. 2 ▸ *a*) since no cross peaks are observed in the ^1^H–^13^C spectra presented in Figs. 4 ▸(*b*) and 4 ▸(*c*). (*e*) Note that the C7—H1 and C6—H4 cross peaks due to longer-range C⋯H proximities cannot be distinguished from the C9—H1 and C12—H4 cross peaks due to one-bond C—H connectivities – in the stick spectrum in Fig. 2 ▸(*c*), open bars denote the calculated (GIPAW) C7 and C6 ^13^C chemical shifts.

**Table 3:** Comparison of experimental ^1^H chemical shifts with calculated^*a*^ (GIPAW) values (all in ppm) for the DI–PM cocrystal for the full crystal structure and an isolated di­thia­non or pyrimethanil mol­ecule

Atom	δ_exp _	δ_crystal_	δ_mol­ecule_	Δδ_crystal–mol­ecule_
H1	7.4	7.4	7.8	−0.4
H2	6.2	5.9	7.4	−1.5
H3	7.7	7.6	7.4	0.2
H4	8.2	8.5	7.8	0.7
H17	9.1	9.7	9.2	0.5
H18	7.7	7.7	7.0	0.7
H19	7.8	7.3	6.6	0.7
H20	7.4	7.6	7.0	0.6
H21	8.0	8.4	6.4	2.0
H22/23/24^*b*^	1.9	1.8	1.9	−0.1
H25	4.0	3.4	6.1	−2.7
H26/27/28^*b*^	2.0	2.0	1.8	0.2
H29	9.1	10.5	6.9	3.6

Notes: (*a*) calculated isotropic chemical shieldings are determined from calculated chemical shieldings according to δ_calc_ = σ_ref_ − σ_calc_, where σ_ref_ equals 30.0 ppm; (*b*) for CH_3_ groups, the calculated ^1^H chemical shifts correspond to the average over the three H atoms.

**Table 4:** Comparison of calculated (GIPAW) NMR chemical shieldings (in ppm) for the DI–PM cocrystal for the full crystal structure and an isolated di­thia­non or pyrimethanil mol­ecule

Atom	σ_mol­ecule_	σ_crystal_	σ_crystal–mol­ecule_
N1	−106.4	−88.9	17.5
N2	−107.2	−88.9	18.3
N9	98.9	91.4	−7.4
N10	−30.1	−33.0	−2.9
N11	−44.5	−42.4	2.1
O1	−363.4	−265.9	97.5
O2	−345.3	−322.2	23.1
S1	330.7	305.6	−25.1
S2	333.8	320.6	−13.2
