# Supplementary material for: Single-crystal X-ray diffraction and NMR crystallography of a 1:1 cocrystal of di­thia­non and pyrimethanil
Source: Acta Crystallogr C Struct Chem. 2017 Feb 6;73(Pt 3):149–56. doi: 10.1107/S2053229617000870 (PMC5391860; doi:10.1107/S2053229617000870)
Supplement: Supplementary file 5 [file c-73-00149-sup5.pdf]

STRUCTURAL CHEMISTRY

Volume 73 (2017)

Supporting information for article:

Single-crystal X-ray diffraction and NMR crystallography of a 1:1 cocrystal of dithianon and pyrimethanil

Ann-Christin Pöppler, Emily K. Corlett, Harriet Pearce, Mark P. Seymour, Matthew Reid, Mark G. Montgomery and Steven P. Brown

Additional Tables (DQ NMR data and distances as well as a comparison of experimental and calculated (GIPAW) ^{13}C chemical shift values) and a Figure showing the difference in the numbering schemes between the crystallographic data and the output of the GIPAW (CASTEP) calculations.

Table S1 ^{1}H DQ correlations[a] (see Fig. 3b) and corresponding H-H distances for the DI-PM co-crystal.

atom 1	δ_{SQ1} (^{1}H) ppm	atom 2	δ_{SQ2} (^{1}H) ppm	δ_{DQ} (^{1}H) ppm	Separation[a] / Å
H1	*7.4*	*H2*	*6.2*	*13.6*	*2.50*
H1	7.4	H23	1.9	9.3	2.54
H1	7.4	H28	2.0	9.4	3.02
H1	7.4	H22	1.9	9.3	3.19
H2	*6.2*	*H3*	*7.7*	*13.9*	*2.47*
H2	6.2	H23	1.9	8.1	2.90
H2	6.2	H24	1.9	8.1	3.03
H2	6.2	H28	2.0	8.2	3.12
H2	6.2	H22	1.9	8.1	3.12
H3	7.7	H26	2.0	9.7	2.45
H3	7.7	H23	1.9	9.6	2.48
H3	*7.7*	*H4*	*8.2*	*15.9*	*2.50*
H3	7.7	H17	9.1	16.8	2.78
H3	7.7	H28	2.0	9.7	3.09
H4	8.2	H24	1.9	10.1	2.56
H4	8.2	H26	2.0	10.2	2.62
H4	8.2	H29	9.1	17.3	2.83
H17	9.1	H26	2.0	11.1	2.48
H17	*9.1*	*H18*	*7.7*	*16.8*	*2.50*
H17	9.1	H27	2.0	11.1	2.72
H17	9.1	H28	2.0	11.1	3.00
H17	9.1	H23	1.9	11.0	3.38
H17	9.1	H21	8.0	17.1	3.42
H18	7.7	H27	2.0	9.7	2.31
H18	*7.7*	*H19*	*7.8*	*15.5*	*2.47*
H18	7.7	H26	2.0	9.7	2.75
H18	7.7	H22	1.9	9.6	2.95
H18	7.7	H20	7.4	15.1	3.38
H18	7.7	H25	4.0	11.7	3.45
H19	7.8	H20	7.4	15.2	2.31
H19	*7.8*	*H20*	*7.4*	*15.2*	*2.51*
H19	7.8	H19	7.8	15.6	3.23
H19	7.8	H25	4.0	11.8	3.24

H19	7.8	H22	1.9	9.7	3.34
H20	*7.4*	*H21*	*8.0*	*15.4*	*2.48*
H20	7.4	H27	2.0	9.4	2.97
H20	7.4	H25	4.0	11.4	3.38
H21	*8.0*	*H29*	*9.1*	*17.1*	*2.21*
H21	8.0	H27	2.0	10.0	2.61
H21	8.0	H28	2.0	10.0	3.24
H22	*1.9*	*H24*	*1.9*	*3.8*	*1.78*
H22	*1.9*	*H23*	*1.9*	*3.8*	*1.78*
H22	*1.9*	*H25*	*4.0*	*5.9*	*2.44*
H22	1.9	H26	2.0	3.9	3.11
H23	*1.9*	*H24*	*1.9*	*3.8*	*1.77*
H23	1.9	H28	2.0	3.9	2.29
H23	1.9	H26	2.0	3.9	3.24
H24	1.9	H29	9.1	11.0	2.64
H24	1.9	H26	2.0	3.9	3.04
H24	1.9	H28	2.0	3.9	3.28
H24	*1.9*	*H25*	*4.0*	*5.9*	*3.36*
H25	*4.0*	*H27*	*2.0*	*6.0*	*2.62*
H25	*4.0*	*H28*	*2.0*	*6.0*	*3.00*
H26	*2.0*	*H28*	*2.0*	*4.0*	*1.78*
H26	*2.0*	*H27*	*2.0*	*4.0*	*1.79*
H27	*2.0*	*H28*	*2.0*	*4.0*	*1.76*

[a] Intramolecular proximities are shown in italics.

Table S2 Comparison of experimental ^{13}C chemical shifts with calculated (GIPAW) values (all in ppm) for the DI-PM co-crystal for the full crystal structure and an isolated dithianon or pyrimethanil molecule.

Atom	δ_{exp}	$\delta_{crystal}$	$\delta_{molecule}$	$\delta_{crystal - molecule}$
C1	114.4	113.8	118.8	–5.0
C2	114.4	115.5	117.9	–2.4
C3	141.4	139.7	141.4	–1.7
C4	131.1	130.1	139.4	–9.3
C5	176.5	179.7	181.9	–2.2
C6	129.8	128.6	127.0	1.6
C7	125.7	126.8	126.8	0.0
C8	178.2	179.9	180.7	–0.8
C9	125.7	126.7	125.0	1.7
C10	133.9	132.6	133.4	–0.8
C11	136.8	139.2	133.7	5.5
C12	129.8	128.5	124.7	3.8
C13	114.4	115.9	113.0	2.9
C14	114.4	114.5	112.5	2.0
C57	141.5	138.5	138.4	0.1
C58	119.4	120.1	114.5	5.6
C59	131.2	131.5	126.9	4.6
C60	130.2	129.3	117.8	11.5
C61	127.7	127.7	124.8	2.9
C62	120.3	120.2	114.1	6.1
C63	160.1	155.5	156.7	–1.2
C64	168.2	168.4	166.9	1.5
C65	23.9	15.3	11.2	4.1
C66	112.6	111.5	106.9	4.6
C67	168.2	168.2	166.9	1.3
C68	25.7	17.2	10.9	6.3

[a] Calculated isotropic chemical shifts are determined from calculated chemical shieldings according to $\delta_{calc} = \sigma_{ref} - \sigma_{calc}$, where σ_{ref} equals 163.2 ppm.

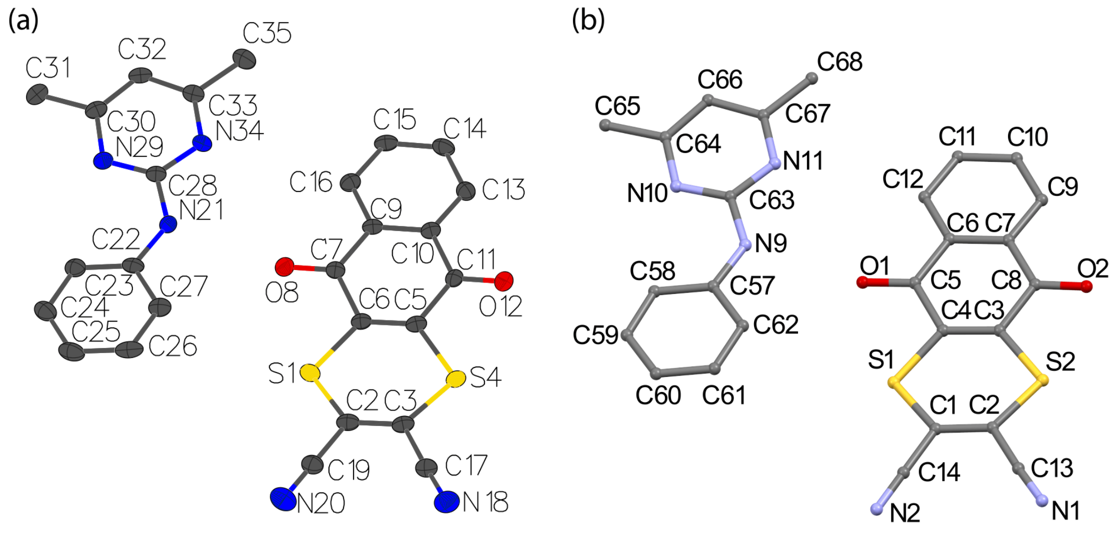

Figure S1 Comparison for the DI-PM co-crystal of numbering in the (a) crystallographic cif file (ellipsoids are shown) and (b) the CASTEP output file. The numbering in (b) is employed in this paper. In (a), anisotropic displacement parameters are depicted at the 50% probability level and hydrogen atoms are omitted for clarity.
